# Improvement of Electrical Transport Performance of BiSbTeSe_2_ by Elemental Doping

**DOI:** 10.3390/ma18051110

**Published:** 2025-02-28

**Authors:** Peng Zhu, Xin Zhang, Liu Yang, Yuqi Zhang, Deng Hu, Fuhong Chen, Haoyu Qi, Zhiwei Wang

**Affiliations:** 1 Centre for Quantum Physics, Key Laboratory of Advanced Optoelectronic Quantum Architecture and Measurement (MOE), School of Physics, Beijing Institute of Technology, Beijing 100081, China; 2 Beijing Key Lab of Nanophotonics and Ultrafine Optoelectronic Systems, Beijing Institute of Technology, Beijing 100081, China; 3 Material Science Center, Yangtze Delta Region Academy of Beijing Institute of Technology, Jiaxing 314011, China; 4 International Centre for Quantum Materials, Beijing Institute of Technology, Zhuhai 519000, China

**Keywords:** BiSbTeSe_2_ topological insulator, chemical doping, carrier density, mobility

## Abstract

A topological insulator with large bulk-insulating behavior and high electron mobility of the surface state is needed urgently, not only because it would be a good platform for studying topological surface states but also because it is a prerequisite for potential future applications. In this work, we demonstrated that tin (Sn) or indium (In) dopants could be introduced into a BiSbTeSe_2_ single crystal. The impacts of the dopants on the bulk-insulating property and electron mobility of the surface state were systematically investigated by electrical transport measurements. The doped single crystals had the same crystal structure as the pristine BiSbTeSe_2_, no impure phase was observed, and all elements were distributed homogeneously. The electrical transport measurements illustrated that slight Sn doping could improve the performance of BiSbTeSe_2_ a lot, as the longitudinal resistivity (*ρ*_xx_), bulk carrier density (*n*_b_), and electron mobility of the surface state (*μ*_s_) reached about 11 Ωcm, 7.40 × 10^14^ cm^−3^, and 6930 cm^2^/(Vs), respectively. By comparison, indium doping could also improve the performance of BiSbTeSe_2_ with *ρ*_xx_, *n*_b_, and *μ*_s_ up to about 13 Ωcm, 1.29 × 10^15^ cm^−3^, and 4500 cm^2^/(Vs), respectively. Our findings suggest that Sn- or indium-doped BiSbTeSe_2_ crystals should be good platforms for studying novel topological properties, as well as promising candidates for low-dissipation electron transport, spin electronics, and quantum computing.

## 1. Introduction

The topological insulator (TI) comprises quantum matter with an insulating bulk state and a metallic surface state that is protected by time-reversal symmetry [1,2]. The surface state hosts many interesting quantum phenomena, including spin momentum locking, Shubnikov–de Haas (SdH) oscillations, the quantum Hall effect (QHE), and so on. The two half-integer QHE of the top and bottom surface states contributes to the integer QHE in three-dimensional (3D) TIs. The Bi_2_Se_3_ material system was predicted to be an ideal 3D TI [3], which was subsequently confirmed by experiments [4,5]. However, due to the formation of intrinsic defects (such as anti-site defects or vacancies) during the process of crystal growth, the bulk state often exhibits a metallic or poor bulk-insulating behavior. Therefore, both surface and bulk states ordinarily contribute to the total conductivity, which makes it difficult to observe the peculiar properties (such as QHE) of the surface state in 3D Tis [6,7,8].

In order to obtain a bulk-insulating property in 3D TIs, one of the strategies that can be applied is element doping, which can dramatically reduce the anti-site defects and vacancies in Bi_2_Se_3_ material systems. The most important achievements are Bi_2_Se_3-*x*_Te*_x_* and Bi_2-*x*_Sb*_x_*Te_3_, in which charge carriers are reciprocally compensated [9,10,11,12]. For example, in Bi_2_Te_2_Se, a ternary TI material with ordered Te-Bi-Se-Bi-Te quintuple layers, bulk resistivity reaches 6 Ωcm at low temperatures [13]. In addition, a noticeable quantum oscillation originating from the topological surface state has been observed in the magnetic field dependence of resistivity, indicating that Bi_2_Te_2_Se is an intrinsic TI with a high surface mobility (~2800 cm^2^/(Vs)) [13,14]. In addition, angle-resolved photoemission spectroscopy (ARPES) measurements have revealed a clear Dirac point located at $\bar{\Gamma }$ point below 0.3 eV of the Fermi surface [15]. Although chemical composition can be finely adjusted to decrease the defects, there are still some questions regarding Bi_2_Te_2_Se: for example, the bulk carrier density at low temperatures is not low enough, and the Dirac point is hidden inside two nearby valence bands. Therefore, the tetradymite solid solution Bi_2-*x*_Sb*_x_*Te_3-*y*_Se*_y_* was proposed to resolve these questions [16]. Since the two predominant carriers with opposite signs induced by (Bi, Sb)/Te anti-site and Se vacancies can be balanced by tuning the ratios of Bi/Sb and Te/Se, Bi_2-*x*_Sb*_x_*Te_3-*y*_Se*_y_* displays a lower carrier density, especially for the composition of *x* = 1 and *y* = 2, i.e., BiSbTeSe_2_ [17,18]. Subsequently, ARPES measurements revealed a linear Dirac cone, which was closest to the Fermi surface in the bulk bandgap compared to other compositions [15]. Furthermore, QHE has been observed from electrical transport measurement and confirmed to originate from the topological surface state in the intrinsic TI of BiSbTeSe_2_ [19]. In a thin BiSbTeSe_2_ sample with its thickness reduced to less than 100 nm, the surface state still dominates the transport properties even at a temperature close to room temperature [20]. K-B Han et al. further improved the quality of BiSbTeSe_2_ single crystals through a two-step melting–Bridgman growth method, achieving a surface mobility of 4400 cm^2^/Vs [21].

Recently, P. Mal et al. investigated the 2D transport of Bi_2-*x*_Sb*_x_*Te_3-*y*_Se*_y_* doped with Sn and In [22] but with a poor bulk-insulating property in their single crystals. Thus, how to obtain high-quality single crystals with excellent bulk-insulating properties and high electron mobility of the surface state is still an unresolved matter. In this work, we carried out single-crystal growth, characterization, and transport measurements of BiSbTeSe_2_, Sn*_x_*Bi_1-*x*_SbTeSe_2_, and In*_y_*Bi_1-*y*_SbTeSe_2_ (*x*, *y* = 0.02, 0.03, 0.04, and 0.05). We found that the performance of the BiSbTeSe_2_ single crystal was successfully improved in some doped samples due to the significant enhancement of the bulk-insulating property and surface mobility, probably making Sn*_x_*Bi_1-*x*_SbTeSe_2_ a valuable material candidate for the potential application of topological insulators in the future.

## 2. Method

High-quality single crystals of BiSbTeSe_2_, Sn*_x_*Bi_1-*x*_SbTeSe_2_, and In*_y_*Bi_1-*y*_SbTeSe_2_ were grown by a two-step melting–Bridgman method. Before growth, the raw materials of Sn (or In), Sb, Se shots, Bi, and Te blocks (99.999%, Alfa Aesar, Shanghai, China) needed to be cleaned to remove the possible oxide layer that formed upon contact with air. They were sealed into quartz tubes filled with 0.8 atm H_2_, individually, and then annealed at a temperature 50 K below their melting points for 10 h, as mentioned in our previous work [23]. Then, 6 g of mixtures of the raw materials was weighed carefully according to the stoichiometric ratio and put into bottom-pointed quartz tubes (inner diameter of 8 mm); this procedure was carried out in an argon-filled glovebox (with H_2_O < 0.1 ppm and O_2_ < 0.1 ppm). The quartz tubes were sealed under a vacuum of 5 × 10^−4^ Pa and then put into a box furnace. Subsequently, the furnace was heated to 850 °C over 6 h and held at this temperature for 48 h. During this stage, the tubes were shaken intermittently to ensure a homogenous melt. After the tubes were slowly cooled down to room temperature, they were transferred into a Bridgman furnace with the temperature settings of the upper and lower zones of 720 °C and 550 °C, respectively. The travel speed of the tube was set to 1 mm/h. Finally, centimeter-scale Sn*_x_*Bi_1-*x*_SbTeSe_2_ and In*_y_*Bi_1-*y*_SbTeSe_2_ shiny single crystals were obtained.

The crystal structures of the as-grown single crystals were characterized by X-ray diffraction (XRD) measurements using a Bruker D2 Phaser (Bruker, Karlsruhe, Germany) diffractometer equipped with Cu-Kα radiation. The chemical compositions of the single crystals were confirmed by energy-dispersive spectroscopy (EDS, JEOL, JSM-IT700HR, Tokyo, Japan). More than two regions were randomly selected for the EDS analysis. The area of each clean-cleaved surface was more than 100 × 100 μm^2^. Elemental mapping was carried out to demonstrate the homogenous distribution of the elements for Sn*_x_*Bi_1-*x*_SbTeSe_2_ and In*_y_*Bi_1-*y*_SbTeSe_2_ single crystals. The electrical transport measurements were conducted on the Physical Property Measurement System (PPMS, Quantum Design, San Diego, CA, USA) equipped with a magnetic field up to 14 T and a temperature down to 1.8 K. Electrical contacts were made in a standard five-terminal configuration using 30 μm thick gold wires attached with silver paint, which could measure longitudinal resistivity and transverse resistivity simultaneously.

## 3. Results

**Figure 1:**
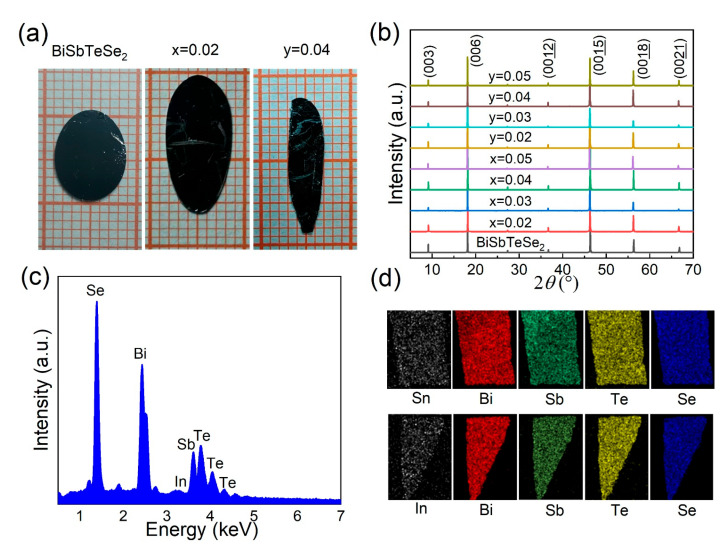
(**a**) Optical images of BiSbTeSe_2_, Sn*_x_*Bi_1-*x*_SbTeSe_2_ (*x* = 0.02), and In_y_Bi_1-*y*_SbTeSe_2_ (*y* = 0.04) single crystals. (**b**) XRD patterns for all as-grown samples, indicating (00*l*) planes. (**c**) Spectrum of EDS for *y* = 0.05 sample. (**d**) Elemental mapping of Sn*_x_*Bi_1-*x*_SbTeSe_2_ (*x* = 0.05) and In*_y_*Bi_1-*y*_SbTeSe_2_ (*y* = 0.05) single crystals.

The parent BiSbTeSe_2_ had a rhombohedral structure with a space group of $R\bar{3}m$ (No. 166). It had ordered Bi/Sb-Te/Se(2)-Se(1)-Te/Se(2)-Bi/Sb quintuple stacking layers with Se(1) fixed in the middle layer and Bi and Sb randomly occupied, like Te and Se(2), as described in the literature [16]. Figure 1a shows optical images of the BiSbTeSe_2_, Sn_0.02_Bi_0.98_SbTeSe_2_, and In_0.04_Bi_0.96_SbTeSe_2_ single crystals that were cleaved from the pristine crystals. One can clearly see that these single crystals are very large, with a size reaching the centimeter scale. The crystal structure of BiSbTeSe_2_, Sn*_x_*Bi_1-*x*_SbTeSe_2_, and In*_y_*Bi_1-*y*_SbTeSe_2_ was confirmed by XRD measurements, and the results are shown in Figure 1b. Only (00*l*) peaks appear in the XRD patterns, which is consistent with what is reported in previous research [21], indicating that the doped single crystals have the same crystal structure as the parent BiSbTeSe_2_, without any impure phase.

In order to check the chemical composition and elemental distribution of Sn*_x_*Bi_1-*x*_SbTeSe_2_ and In*_y_*Bi_1-*y*_SbTeSe_2_, EDS analysis was conducted on an SEM equipped with an X-ray spectrometer. Figure 1c illustrates the EDS results of In_0.05_Bi_0.95_SbTeSe_2_: one can clearly see the indium peak except for peaks of other four elements, which confirms that indium was successfully doped in the BiSbTeSe_2_ crystal. The actual content of elements for all crystals is listed in Table 1. It is clear that the ratio of Se and Te is quite close to 2:1 for most of the crystals; however, the actual ratio of Bi to Sb is much higher than 1:1, even for the parent BiSbTeSe_2_, which means that there are always anti-site defects of Sb occupied by Bi. For the *x* = 0.05 and *y* = 0.05 samples, the EDS results revealed that the actual contents were about 1.2% and 3.2%, respectively. Notably, the contents of the doping elements (i.e., Sn and In) in our samples with lower nominal composition could not be effectively detected due to the doping concentration being lower than the instrument limit. Nonetheless, we believe that Sn and In were successfully doped in BiSbTeSe_2_ based on the electrical transport results discussed below. For convenience, we will still use the nominal compositions for our discussion below. Elemental mappings were performed to further display the distribution of all elements, as shown in Figure 1d, which suggests a clear homogeneous distribution for all the elements.

**Figure 2:**
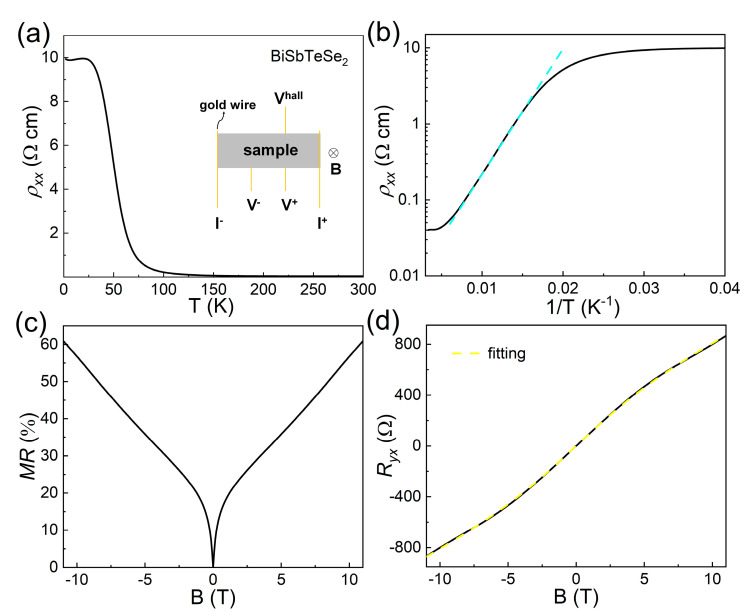
Electrical transport results of parent BiSbTeSe_2_ bulk crystal. (**a**) Temperature dependence of resistivity of BiSbTeSe_2_. The inset shows the measurement scheme. (**b**) Arrhenius plots of *ρ_xx_*(*T*). The cyan dashed line represents the linear fitting. (**c**) Magnetic field dependence of MR measured at 2 K. (**d**) Hall resistance measured at 2 K; the yellow dashed curve shows the result of two-band fitting.

Electrical transport measurements of parent and doped BiSbTeSe_2_ samples were conducted to investigate the doping effect on the bulk-insulating property and electron mobility of the surface state. Figure 2a shows the temperature dependence of longitudinal resistivity $\rho_{xx}$ in the parent BiSbTeSe_2_. With the temperature decreasing, $\rho_{xx}$ increased slowly in the range from 300 K to 150 K and, later, rapidly from 150 K to 30 K. An almost saturated behavior was observed when the temperature was lower than 30 K, and the *ρ_xx_* reached 9.8 Ωcm at 2 K. It was thus clear that a bulk-insulating property had been achieved in our BiSbTeSe_2_ single crystal. The saturated behavior below 30 K originated from the surface state-dominated transport, while the bulk was deeply suppressed, which was consistent with previous results [17,19].

**Figure 3:**
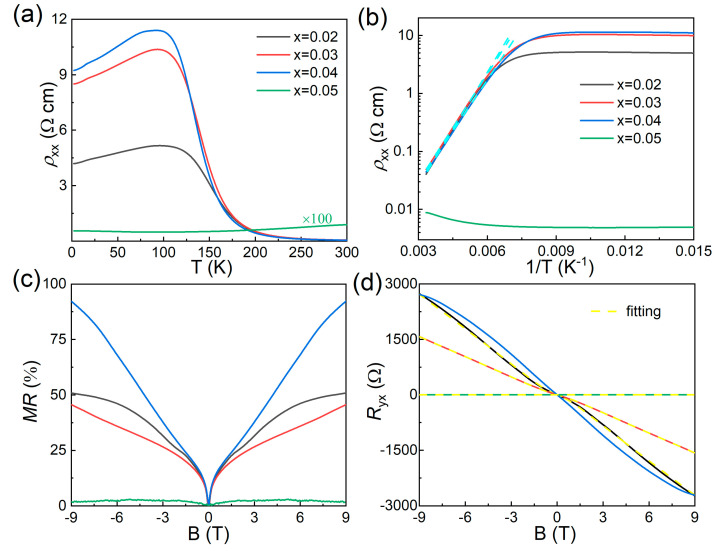
Electrical transport results of Sn*_x_*Bi_1-*x*_SbTeSe_2_ crystals: (**a**) temperature dependence of resistivity of Sn*_x_*Bi_1-*x*_SbTeSe_2_; (**b**) Arrhenius plots of *ρ_xx_*(*T*), the cyan dashed line represents the linear fitting; (**c**) MR at 2 K; and (**d**) Hall resistance at 2 K, where the yellow curves show the respective two-band fitting.

**Figure 4:**
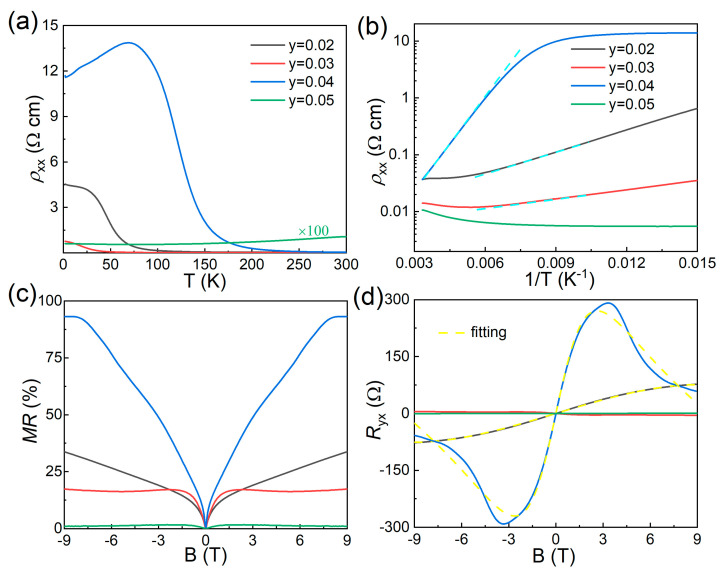
Electrical transport results of parent In_*y*_Bi_1-*y*_SbTeSe_2_ crystals: (**a**) temperature dependence of resistivity curves *ρ*_xx_(*T*); (**b**) Arrhenius plots of *ρ_xx_*(*T*), the cyan dashed line represents the linear fitting; (**c**) MR at 2 K; and (**d**) Hall resistance at 2 K, where the yellow curves show the respective two-band fitting.

A similar bulk-insulating behavior was also observed in the Sn*_x_*Bi_1-*x*_SbTeSe_2_ samples with *x* from 0.02 to 0.04. The bulk-insulating behavior began to appear at 250 K in the Sn-doped BiSbTeSe_2_ rather than 125 K in the parent BiSbTeSe_2_, and the largest *ρ_xx_* could reach about 11 Ωcm at around 100 K in the *x* = 0.04 sample, as seen in Figure 3a, which suggests that better bulk-insulating properties can be achieved via Sn doping in BiSbTeSe_2_. However, the situation became worse in the *x* = 0.05 sample, as we could see a metallic behavior in the high-temperature region and a poor bulk-insulating behavior in the low-temperature region, and the magnitude of *ρ_xx_* at 2 K was almost 100 times smaller than in the other Sn-doped crystals. An improvement in the bulk-insulating behavior was also observed in the In*_y_*Bi_1-*y*_SbTeSe_2_ crystals, especially for the *y* = 0.04 sample, as shown in Figure 4a: the largest *ρ_xx_* could reach about 13 Ωcm at around 75 K. This was the best bulk-insulating behavior, meaning that there were less defects in these samples, which suggested that Sn or In was effectively doped in BiSbTeSe_2_. The *ρ_xx_*(*T*) curves, which exhibited a thermally activated behavior, could be fitted by Arrhenius law,

(1) $$\rho_{xx}\sim exp\left(\frac{\Delta }{k_{B}T}\right)$$

where $k_{B}$ is the Boltzmann constant, and Δ is the thermal activation energy. The cyan dashed line represents the linear fitting of the high-temperature region, as shown in Figure 2b, Figure 3b, and Figure 4b. Here, we ignored the linear fittings of samples of *x* = 0.05 and *y* = 0.05 due to their poor bulk-insulating behavior. The calculated Δ values are listed in Table 2, with the largest value reaching about 150 meV. The values of Δ in the single crystals with excellent bulk-insulating properties were almost three times larger than that in pristine BiSbTeSe2 whose value is about 38 meV. These large values of Δ were almost consistent with half of the bulk bandgap in BiSbTeSe_2_ (300 meV) measured by ARPES [15], a signature of the intrinsic nature of semiconducting Sn*_x_*Bi_1-*x*_SbTeSe_2_ and In*_y_*Bi_1-*y*_SbTeSe_2_ crystals, indicating that the charge carrier in these samples were well compensated.

A weak antilocalization (WAL) effect is considered a typical indication of an intrinsic TI. Samples with excellent bulk-insulating behaviors always exhibited WAL, featuring the appearance of V-shaped cusps at a low magnetic field, as shown in Figure 2c, Figure 3c, and Figure 4c, together with the observation of positive magnetoresistance (MR) when the magnetic field was applied perpendicular to the *ab* plane. Unsaturated linear MR was observed in the parent BiSbTeSe_2_ sample at a high magnetic field up to 11 T, and the highest MR reached about 60%; this unsaturated linear MR behavior was similar to that reported elsewhere [22]. For the doped samples with excellent bulk-insulating properties, the MR showed a nonlinear behavior under a highly magnetic field regime, and the highest MR reached about 90%. On the other hand, for samples of *x* = 0.05 and *y* = 0.05, the MR was negligible.

Figure 2d, Figure 3d, and Figure 4d show the magnetic field-dependent Hall resistance ($R_{yx}$) for the parent, Sn-doped, and In-doped BiSbTeSe_2_ samples, respectively, which were measured at 2 K. It is evident that the *R_yx_*-B curves for the samples with excellent bulk-insulating properties exhibited a nonlinear behavior, indicating that there were carriers from at least two bands which contributed to the transport results in these samples. The BiSbTeSe_2_ sample showed a positive slope while all Sn-doped samples except for *x* = 0.05 showed negative slopes, illustrating that the main carrier in the BiSbTeSe_2_ sample was the hole (*p*-type), while in the Sn-doped samples (except for *x* = 0.05), it was an electron (*n*-type). The bulk-insulating samples always had a large *R_yx_*, meaning that the dominated carrier density should have been smaller than that in the samples with poor bulk-insulating abilities. In order to quantitatively analyze the carrier density and electron mobility of the surface state, the observed *R_yx_*-B curves were fitted using a two-band model,

(2) $$R_{yx}=-\frac{B}{e}\cdot \frac{(n_{1}\mu_{1}^{2}+n_{2}\mu_{2}^{2})+B^{2}\mu_{1}^{2}\mu_{2}^{2}\left(n_{1}+n_{2}\right)}{{\left(\left|n_{1}\right|\mu_{1}+\left|n_{2}\right|\mu_{2}\right)}^{2}+B^{2}\mu_{1}^{2}\mu_{2}^{2}{\left(n_{1}+n_{2}\right)}^{2}}$$

where the *B*, *e*, $n_{1}$, $n_{2}$, $\mu_{1},$ and $\mu_{2}$ represent the magnetic field, charge of electron, carrier density of the surface and bulk, and electron mobility of the surface and bulk, respectively. The yellow curves give the fitting results, and the extracted values of $n_{1}$, $n_{2}$, $\mu_{1},$ and $\mu_{2}$ are listed in Table 2 for all measured samples. The lower *n*-type carrier density derived from the surface state (*n*_1_) was as low as 10^10^ cm^−2^ orders for the excellent bulk-insulating Sn-doped BiSbTeSe_2_ samples, which was one order lower than that for BiSbTeSe_2_. In addition, the optimized samples (*x* = 0.02 and 0.03) also displayed a lower 2D bulk carrier density *n*_2_ compared to BiSbTeSe_2_ [20]. One could easily transfer the 2D carrier density *n*_2_ to a 3D one by dividing the sample thickness *t*, giving a 3D bulk carrier density equal to 1.50 × 10^15^, 1.38 × 10^15^, 7.40 × 10^14^, and 4.3 × 10^15^ cm^−3^ for the parent BiSbTeSe_2_, *x* = 0.02, 0.03, and *y* = 0.04 samples, respectively. Notably, the *x* = 0.02 sample had the lowest carrier density. At the same time, the electron mobility of the surface state could be calculated to be about 6930 cm^2^/(Vs) for the *x* = 0.02 sample, which was more than five times that in our BiSbTeSe_2_ sample, i.e., 1324 cm^2^/(Vs), also higher than the highest value reported in BiSbTeSe_2_ [4400 cm^2^/(Vs)] [19,21]. The surface mobility for the other samples is listed in Table 2, where we can find that the samples with a larger activated energy Δ usually had a higher surface mobility. We also would like to point out that the fitting results of the *x* = 0.04, *y* = 0.03, and *y* = 0.05 samples were not successful in obtaining the carrier density and mobility.

It has been previously reported that Sn and In are special impurities that form a resonant level within the bandgap, acting as charge buffers [9], and the resonant level in BiSbTeSe_2_ would lead to the observed low carrier density in bulk and the high surface electron mobility on the surface. However, there are some different effect between tin doping and indium doping. For example, compared to the undoped BiSbTeSe_2_, Sn doping not only changed the sign of the bulk carrier type but also lowered the carrier density in the best bulk-insulating samples, i.e., the *x* = 0.02 and 0.03 samples. On the other hand, indium doping kept the bulk carrier type the same and made the carrier density a bit higher. This suggests that tin is probably in a +4 valance state and acts as a donor, while indium is probably in a +1 state and an acceptor.

## 4. Conclusions

In summary, we systematically investigated the effect of chemical doping on the physical properties, including the bulk-insulating behavior, carrier density, and surface mobility, in Sn- and In-doped BiSbTeSe_2_. The results indicated that slight Sn or In doping could significantly enhance the surface mobility, with the highest value reaching 6930 cm^2^/(Vs) in the *x* = 0.02 sample, and reduce the 3D bulk carrier density to an order of 10^15^ cm^−3^ at the same time. The achievement of an intrinsic topological insulator in both Sn- and In-doped BiSbTeSe_2_, with excellent bulk-insulating properties and high surface mobility, should have remarkable potential applications in microelectronics, optoelectronics, and spintronics in the future.

## Figures and Tables

**Table 1:** Actual compositions of BiSbTeSe_2_, Sn*_x_*Bi_1-*x*_SbTeSe_2_, and In*_y_*Bi_1-*y*_SbTeSe_2_ single crystals.

Nominal Samples		Actual Contents (%)			
		Bi	Sb	Te	Se
BiSbTeSe_2_		24.88	17.13	19.68	38.31
*x*	0.02	24.84	15.63	18.90	40.63
	0.03	23.43	17.62	19.56	39.38
	0.04	24.14	17.58	20.10	38.18
	0.05	24.37	17.57	20.30	37.76
*y*	0.02	22.43	19.12	21.07	37.38
	0.03	24.19	17.81	20.04	37.97
	0.04	23.13	18.77	20.96	37.07
	0.05	21.84	19.49	21.07	36.83

**Table 2:** Important parameters of Sn*_x_*Bi_1-*x*_SbTeSe_2_ and In*_y_*Bi_1-*y*_SbTeSe_2_. The values of carrier density and mobility were extracted from a two-band model, except for *y* = 0.05. The *n*_1_ and *μ*_1_ stand for the carrier density and mobility from the surface. The *n*_2_ and *μ*_2_ stand for the carrier density and mobility from the bulk. The positive (negative) values stand for *n*-type and *p*-type carriers, respectively. The thermal activation energies (Δ) were extracted from *ρ_xx_*(*T*).

Nominal Samples		Carrier Density and Mobility				*n*_b_ (cm^−3^)	Δ (meV)
		*n*_1_ (cm^−2^)	*μ*_1_ (cm^2^V^−1^s^−1^)	*n*_2_ (cm^−2^)	*μ*_1_ (cm^2^V^−1^s^1^)		
BiSbTeSe_2_		−4.15 × 10^11^	1324	−9.16 × 10^12^	252	−1.50 × 10^15^	38
*x*	0.02	−2.10 × 10^10^	6930	2.07 × 10^12^	1193	1.38 × 10^15^	153
	0.03	−2.44 × 10^10^	5573	3.55 × 10^12^	944	7.40 × 10^14^	144
	0.04	-	-	-	-	-	139
	0.05	8.87 × 10^14^	675	−3.62 × 10^16^	431	−2.55 × 10^18^	
*y*	0.02	1.12 × 10^13^	2182	−1.29 × 10^13^	1257	−1.29 × 10^15^	30
	0.03	-	-	-	-	-	13
	0.04	2.53 × 10^11^	4575	−3.25 × 10^13^	89	−4.33 × 10^15^	125
	0.05					−1.26 × 10^18^	

## Data Availability

The original contributions presented in this study are included in the article. Further inquiries can be directed to the corresponding author.
